# Decolonisation of curricula in undergraduate dental education: an exploratory study

**DOI:** 10.1038/s41415-022-4923-1

**Published:** 2022-09-09

**Authors:** Kamran Ali, Jennie Winter, Oliver Webb, Daniel Zahra

**Affiliations:** 4141510519001 Qatar University, Doha, Qatar; Faculty of Health, Plymouth University, Plymouth, UK; 4141510519002 Plymouth Marjon University, Plymouth, UK; 4141510519003 Educational Development, University of Plymouth, Plymouth, UK

## Abstract

**Aims** To explore experiences and perceptions of students and staff regarding decolonisation of the curriculum in a dental undergraduate programme.

**Methods ** Participants were invited to respond to an online survey on decolonisation of the dental curriculum. The target population included current students on the Bachelor of Dental Surgery and Bachelor of Dental Therapy and Hygiene programmes, as well as dental staff at a university in the South West region of England. The common items for student and staff versions of the survey were focused on six themes: representation; content; peer engagement; assessment; language and communication; and culture. All responses were anonymous. Data on programme, year of study, age, sex and ethnicity were captured on a voluntary basis.

**Results** In total, 34 staff members and 120 students from two different programmes participated in the survey, yielding a response rate of 87.17% for staff and 45.28% for students. A comparison showed that average student responses were lower compared with average staff responses. Of the 24 survey items, 17 showed significantly lower scores reported by minority ethnic (ME) students. ME students were, when compared with white counterparts, less likely to report that their programme included opportunities for group discussions about ethnicity and privilege. Similar comparisons of staff responses did not show significant differences between white and ME staff. Nevertheless, responses by staff and students across the board highlighted the need for further steps to improve the representation of ME groups in the curriculum.

**Conclusions** This study provides useful insights into the perceptions and experiences of students and staff regarding the decolonisation of the dental curriculum in an undergraduate dental programme. Responses by the participants across the board identified several areas which could benefit from better representation of ME groups. Significant differences were noted between staff and student scores and also between white and ME students, indicating the latter group demonstrated more awareness regarding issues of representation. The findings underscore the need to take further steps to decolonise dental curricula.

## Key points

- Explains the rationale for the decolonisation of educational curricula in higher education.
- Reports perceptions and experiences of students and staff regarding decolonisation of the dental curriculum at a British university.
- Underscores the need for further actions to encourage better representation of minority ethnic groups in dental education.

## Introduction

Colonisation is a historic and global phenomenon stemming from the subjugation of Indigenous populations by a small number of European governments in the fifteenth century. By the nineteenth century, more than 80% of countries and 750 million people worldwide lived under colonial rule. However, continued challenges to colonial empire, the advent of world war and the creation of the United Nations instigated a sustained period of international decolonisation.^1^^,^^2^ Decolonisation began with the physical withdrawal of the colonisers and eventual political dismantling of colonialism, but the legacy of colonialism has persisted and is continually contested. Decolonisation then, as a political and social agenda, remains relevant. Among a range of definitions, Von Bismark (2012) describes decolonisation as 'the reversal of the process of European imperial expansion with all its political, economic, social, cultural and linguistic consequences'.^3^

Although implicit, colonial influences may still persist in universities and shape our assumptions about how society needs to work, what needs to be taught and how it needs to be taught. The quest for non-Eurocentric paradigms triggered a 'decolonising the curriculum' movement at the University of Cape Town to remove influences of colonialism and provide representation to the knowledge and perspectives of Indigenous people. In what became known as the 'Rhodes Must Fall' campaign, students advocated for the removal of the statue of Cecil John Rhodes from the University's campus.^4^ The campaign challenged the Eurocentric hierarchies of race, social class and gender in South Africa. Since then, this movement spread further afield and was mirrored in the UK as the 'Rhodes Must Fall' Oxford campaign. Student bodies and staff in UK universities are actively campaigning for decolonising curricula in higher education (HE) institutions. Student unions at leading universities, including Oxford, Cambridge, London, Kent, Leeds and many more, are already part of this movement.^5^

'Decolonisation' of the curricula is a complex concept which is shaped by different social, political and educational interpretations. More importantly, however, it needs to be viewed as an approach which is contextual to different disciplines and subject areas. Decolonisation of the curricula may be described as the 'creation of spaces and resources for a dialogue among all members of the university on how to imagine and envision all cultures and knowledge systems in the curriculum and with respect to what is being taught and how it frames the world'.^3^ Such discourse requires deconstruction, rethinking and reconstruction in order to make curricula inclusive and representative of different communities, voices and perspectives. A contextual approach to curriculum design and delivery may allow the universities to evaluate how curriculum - and therefore education - influences social relations, practices and culture.^6^ Such steps can help build an education culture where every human being has an equal right to contribute and influence creation of knowledge.

Decolonisation of the curriculum also needs to be viewed in the context of attainment gaps in HE. While there is compelling evidence of an ethnicity attainment gap in HE in the UK, debate continues regarding the cause.^7^ Some evidence suggests the gap is partly structural, attributable to 'disadvantaged' backgrounds, pre-HE experiences and entry qualifications (all arguably caused by the tendrils of colonisation across time).^8^ However, where factors including prior attainment have been controlled for, differences remain between minority ethnic (ME) and white students:^9^ 'controlling for entry qualifications, Black students are between 6 and 28 percentage points less likely than white students to get a higher classification degree, while Asian students are between 3 and 17 percentage points less likely. The differences exist at all levels of entry qualifications, so are even apparent among students who enter higher education with very high prior attainment'.^10^ It appears that inequalities within HE mirror those in wider UK society: 'broader political and social realities are evident on campuses affecting the experiences and actions of staff and students'.^11^ Once in HE, contributing factors, such as relationships with and between staff and students; the curriculum; social, cultural capital; and identity factors, all come into play and there is evidence of inequality remaining through to graduate outcomes, where unemployment for ME groups is double that of those who are white.^12^

While further research is required to evaluate the impact of decolonisation on addressing attainment gaps in HE, racial inequalities have intensified debate on decolonisation in HE. There are growing calls for a nuanced approach to addressing cultural, social and political structures that reinforce and reproduce racially motivated differences in attainment. Potentially, decolonisation can help mitigate the attainment gap by questioning and then transforming these meta-structures to add value to the student experience. It is helpful to distinguish decolonisation from inclusivity. The inclusivity mission is to improve the educational experience for all (including students with protected characteristics) and aims to remove political/power relationships from the curriculum. Conversely, decolonisation seeks to challenge dominant forms of thought and practice and to radicalise, not de-politicalise. The aim of a decolonised curriculum extends beyond the attainment of individual students at a given university, important as that is. Ultimately, it is about transforming society and breaking down structural inequalities and institutional racism. As centres of knowledge production, universities should be at the vanguard of these efforts.^13^

Beyond issues of representation, decolonisation demands deeper critique of the construction and content of the curriculum and the canons of disciplinary knowledge. Decolonisation highlights how knowledge is socially and temporally situated, with the current dominant social paradigm being Eurocentric and rooted in colonial epistemology. Decolonisation of HE involves liberating curricula from these selective narratives, instead providing students with 'diverse academic learning environments, curricula and approaches to research, within which Indigenous cultures, histories and knowledge are embedded'.^14^

## Decolonising the dental curricula

Like other healthcare professions, dentistry has, historically, been conceptualised as a 'white' profession in the UK.^15^ Despite growing representation of ME students in dentistry, white men continue to dominate academic positions. Approximately 76% of academic faculty members identified as white, 9% as Asian and 2% as Black. Moreover, in UK dental schools, the proportion of ME groups in senior posts remains low.^16^ Limited representation of ME groups in institutional power structures and decision-making processes may impact on the educational experiences of ME students, potentially erecting barriers to attainment and career progression.^17^

Dental institutions need to be proactive and take appropriate steps to address racial inequalities in dental education. For example, effective use of platforms such as Health Education England, which commissions dental education in the UK, and the Dental Schools Council, can facilitate sharing the data on student attainment and collaborate to address any gaps.

The Race Equality Charter by Advance HE provides an excellent framework for the HE institutions to identify and self-reflect on institutional and cultural barriers faced by ME staff and students.^18^ Universities in the UK already have a committee structure in place to address issues of equality, diversity and inclusion and these committees can facilitate engagement with ME students and staff. Similarly, lay members on dental committees should include ME group representation. Together with student voice, public representation should be used to inform curriculum development, teaching and assessments. Moreover, the reading lists in dental courses can be revisited by engaging with ME students and staff to ensure that they are inclusive and people from under-represented groups are represented appropriately.^19^

Equally, under-representation of ME groups in healthcare curricula can translate into disparities in patient care for these groups, with far reaching implications for their health and wellbeing, as observed during the COVID-19 pandemic.^20^^,^^21^ It is acknowledged that racial inequalities in dental education are not solely related to curriculum content and require a combination of awareness and knowledge of the ethnic demographics of the local communities to effectively address the oral healthcare needs of culturally and lingually diverse populations.^22^ Biomedical foundations of dental care need to be integrated with social determinants of health to improve the experiences of ME patients.^23^ Dental institutions need to develop faculty resources and effective educational strategies to boost students' knowledge, understanding and skills to achieve a minimum cultural competence standards.^24^

The impact of medical curricula on patient health inequalities and lower attainment among ME students have not been researched adequately.^25^ Nevertheless, calls for decolonisation of dental curricula are growing.^26^^,^^27^ This paper details a survey on decolonisation of the dental curriculum, which explored experiences and perceptions of students and staff at a dental school in South West England.

## Methods

### Development and piloting of the survey instrument

Google searches, utilising the terms 'decolonisation' and 'ac uk', were conducted to identify materials relevant to a UK HE context. No existing survey tool was found. Nonetheless, three comprehensive 'toolkits' were identified at SOAS SOAS University of London, University College London and Kingston University.^28^^,^^29^^,^^30^ Scrutiny of the toolkits indicated consistent themes regarding decolonisation and the curriculum: representation; content; peer engagement; assessment; language and communication; and culture. Synthesising across toolkits, the educational development team developed a draft survey featuring four items for each of these six themes. Staff and student versions were produced featuring subtle wording changes where appropriate.

Ethical approval for the study was granted by the Research Ethics Committee, Plymouth University. The draft survey was piloted, first among staff and student interns attached to the institutional education development team and then among staff (n >40) and students (n >90) in two university schools. Response rate, item completion rate and open comments taken form the surveys indicated good participant comprehension. Following deliberations between the educational development team and dental faculty, an additional item relating to teaching on presentation of skin diseases in ME patients was added to the questionnaires for staff and students. Consequently, the surveys were finalised for use in the dental school (Appendix 1).

### Survey administration

Decolonisation of educational curricula is a complex and evolving concept and many students and faculty staff may not be *au fait* with the rationale and dynamics of decolonisation. To mitigate against the relatively limited understanding, the lead author delivered separate presentations on decolonisation of dental curricula to faculty staff and students and signposted them to relevant resources. Moreover, the participation information sheet accompanying the invites to the participants included a description of decolonisation of dental curricula, scope and purpose of the research and contact details of the research team for any queries by the participants.

In Spring 2021, invites and electronic survey links were emailed to all current Bachelor of Dental Surgery (BDS) students (n = 227) and Bachelor of Dental Therapy and Hygiene (BDTH) students (n = 38), and all dental ataff (n = 39). A reminder was sent to all participants two weeks later. Participants who responded to the invite were required to provide an online consent before recording their responses to the survey questions anonymously. Data on programme, year of study, age, sex and ethnicity were captured on a voluntary basis.

## Results

In total, 34 staff members participated in the study, yielding a response rate of 87.17%. Among staff, 31 reported affiliation with the BDS programme, and three with BDTH. Among the students, 120 responded to the survey, showing an overall response rate of 45.28%. Among students, 89 reported being on the BDS programme (23, 12, 20, 13, and 21, respectively, in Stages 1-5), which translated into a response rate of 39.20%. Finally, 31 BDTH students responded to the survey (10, 12, and 9, respectively, in Stages 1-3), giving a response rate of 81.57%. No information on sex was provided by staff. All 31 BDTH students identified as women. Among the BDS students, 57 identified as women, 30 as men and two preferred not to say. Table 1 summarises the ethnic profiles of the participants.

**Table 1:** Ethnicity of participants

**Group**	**Ethnicity**	**Count**	**Percentage**
**Staff**	Asian/Asian British	1	2.94
	Black/African/Caribbean/Black British	0	0
	Multiracial/multiple ethnic groups	0	0
	Other ethnic group	0	0
	Prefer not to say	3	8.82
	White	30	88.24
**Students**	Asian/Asian British	45	37.5
	Black/African/Caribbean/Black British	12	10
	Multiracial/multiple ethnic groups	5	4.17
	Other ethnic group	7	5.83
	Prefer not to say	1	0.83
	White	50	41.67

All items featured the response options 'not at all', 'to some extent' and 'very much' (scored 1-3, respectively). Items LC1 and CU2 were reverse scored where combined with other items but not where examined individually.

Table 2 provides descriptive statistics for each item, stratified by group. The minimum and maximum scores for each item were 1 and 3, respectively. Alongside mean differences, p-values from chi-squared tests of response profiles are shown. P-values <0.05 (highlighted) indicate statistically significant differences in response profiles between students and staff.

**Table 2:** Descriptive statistics for item responses by staff and student respondents (mean difference and p-values for statistical significance of associations between groups and responses)

**Question**	**Staff (n = 34)**				**Students (n = 120)**				**Mean difference**	**p**
	**Mean**	**Standard deviation (SD)**	**Min**	**Max**	**Mean**	**SD**	**Min**	**Max**		
R1	2.24	0.50	1	3	2.06	0.75	1	3	-0.18	0.007
R2	2.35	0.49	2	3	2.08	0.62	1	3	-0.27	0.043
R3	2.29	0.64	1	3	2.09	0.66	1	3	-0.20	0.336
R4	2.35	0.66	1	3	1.79	0.69	1	3	-0.56	<0.001
C1	1.69	0.54	1	3	1.77	0.68	1	3	0.08	0.165
C2	2.00	0.45	1	3	1.72	0.78	1	3	-0.28	<0.001
C3	1.83	0.59	1	3	1.7	0.63	1	3	-0.13	0.440
C4	2.45	0.57	1	3	2.03	0.60	1	3	-0.42	0.003
PE1	2.79	0.42	2	3	2.6	0.54	1	3	-0.19	0.185
PE2	2.26	0.68	1	3	1.87	0.71	1	3	-0.39	0.027
PE3	2.30	0.65	1	3	1.78	0.79	1	3	-0.52	0.002
PE4	2.13	0.68	1	3	1.66	0.76	1	3	-0.47	0.003
A1	2.00	0.59	1	3	1.68	0.69	1	3	-0.32	0.017
A2	2.13	0.62	1	3	1.69	0.69	1	3	-0.44	0.005
A3	2.42	0.50	2	3	2.1	0.71	1	3	-0.32	0.021
A4	1.93	0.52	1	3	1.49	0.63	1	3	-0.44	<0.001
LC1	1.30	0.53	1	3	1.26	0.53	1	3	-0.04	0.678
LC2	2.28	0.63	1	3	2.03	0.62	1	3	-0.25	0.104
LC3	1.96	0.69	1	3	1.81	0.62	1	3	-0.15	0.373
LC4	2.23	0.63	1	3	1.71	0.67	1	3	-0.52	0.001
CU1	2.25	0.72	1	3	1.83	0.76	1	3	-0.42	0.023
CU2	1.13	0.35	1	2	1.43	0.68	1	3	0.30	0.069
CU3	2.65	0.55	1	3	2.53	0.57	1	3	-0.12	0.530
CU4	2.3	0.60	1	3	1.95	0.74	1	3	-0.35	0.030

Reported p-values are based on 10,000 Markov chain Monte Carlo simulated replicates, to minimise impact of small subgroup/cell sizes, though some categories remain empty.

Of the 24 items given to staff and students, there were significantly different average ratings for 15 (Table 2). Notably, in all cases of significance, average students' responses were lower compared with average staff responses. Looking closely at Table 2, particularly notable disparities appeared to occur in the 'peer engagement' and 'assessment' domains. In both cases, significant differences were identified for all four of the constituent items. Moreover, the scale of the mean differences between staff and student responses appeared particularly large for items under these headings.

Using the same approach to statistical analysis, student responses to each question were analysed further, to compare the scores for white and ME students. The results are depicted in Table 3.

**Table 3:** Descriptive statistics for item responses between white and ME students (mean difference, and p-values for statistical significance of associations between groups and responses)

**Question**	**White**					**ME**					**Mean difference**	**p**
	**n**	**Mean**	**SD**	**Min**	**Max**	**n**	**Mean**	**SD**	**Min**	**Max**		
R1	50	2.69	0.51	1	3	70	1.61	0.55	1	3	-1.08	<0.001
R2	50	2.45	0.5	2	3	70	1.81	0.55	1	3	-0.64	<0.001
R3	50	2.31	0.65	1	3	70	1.94	0.62	1	3	-0.37	0.008
R4	50	2.06	0.78	1	3	70	1.6	0.55	1	3	-0.46	<0.001
C1	50	2.06	0.68	1	3	70	1.57	0.61	1	3	-0.49	<0.001
C2	50	2.02	0.88	1	3	70	1.51	0.63	1	3	-0.51	<0.001
C3	50	1.78	0.67	1	3	70	1.64	0.6	1	3	-0.14	0.411
C4	50	2.12	0.53	1	3	70	1.97	0.65	1	3	-0.15	0.121
PE1	50	2.82	0.39	2	3	70	2.44	0.58	1	3	-0.38	<0.001
PE2	50	2.23	0.67	1	3	70	1.62	0.62	1	3	-0.61	<0.001
PE3	50	2.09	0.88	1	3	70	1.57	0.65	1	3	-0.52	<0.001
PE4	50	2.02	0.83	1	3	70	1.41	0.6	1	3	-0.61	<0.001
A1	50	1.9	0.68	1	3	70	1.52	0.66	1	3	-0.38	0.009
A2	50	1.88	0.7	1	3	70	1.57	0.65	1	3	-0.31	0.058
A3	50	2.1	0.69	1	3	70	2.1	0.74	1	3	0	0.801
A4	50	1.63	0.64	1	3	70	1.4	0.6	1	3	-0.23	0.081
LC1	50	1.14	0.46	1	3	70	1.34	0.56	1	3	0.2	0.019
LC2	50	2.23	0.6	1	3	70	1.88	0.59	1	3	-0.35	0.009
LC3	50	1.91	0.67	1	3	70	1.74	0.59	1	3	-0.17	0.224
LC4	50	1.73	0.67	1	3	70	1.7	0.68	1	3	-0.03	0.933
CU1	50	2.18	0.77	1	3	70	1.58	0.65	1	3	-0.6	<0.001
CU2	50	1.22	0.47	1	3	70	1.57	0.76	1	3	0.35	0.024
CU3	50	2.67	0.56	1	3	70	2.43	0.56	1	3	-0.24	0.011
CU4	50	2.24	0.71	1	3	70	1.74	0.69	1	3	-0.5	0.002

Of the 24 surveys items, 17 showed a significant difference in responses between white and ME students. In all cases of significance, the direction of the scores suggested less favourable responses among ME students. Scrutiny of Table 3 reveals those domains with the most divergent pattern of responses. For the themes of 'representation', 'peer engagement', and 'culture', there were significant differences for all four constituent items and substantial mean differences. In the domains of 'content' and 'language and communication', a mixed picture occurred; white and ME students provided divergent responses for two of the four constituent items. Under 'assessment', significantly different responses were only identified for one item (A1 - 'do assessments in the programme allow participants to draw in personal experiences, including those relating to ethnicity and privilege?').

Positively, it appears that white and ME students report less divergent perspectives around the pivotal domain of 'assessment', which can be so impactful on educational and career outcomes. This outcome does, however, highlight the importance of appraising results from the 'intra-student' analyses (that is, white versus ME students), alongside the staff versus student analyses. Despite the relative consistency of white and ME students' views on 'assessment', student perspectives on this domain were, overall, substantially less favourable compared with all staff (Table 2).

Finally, scores for individual items in each domain were combined to produce a single, average score. These scores were then analysed to establish any significant differences according to group (that is, staff versus students) and ethnicity (that is, white versus ME students). The findings are shown in Table 4.

**Table 4:** Descriptive statistics for responses within each domain, by group, ethnicity (white or minority ethnic group) and group by ethnicity

**Domain**	**Mean**	**SD**	**Min**	**Max**	**Mean**	**SD**	**Min**	**Max**	**Mean difference**	p*
	**Staff (all)**				**Students (all)**				**-**	
Assessment	2.12	0.58	1	3	1.74	0.71	1	3	-0.38	<0.001
Content	1.99	0.6	1	3	1.81	0.69	1	3	-0.18	0.004
Culture	2.51	0.62	1	3	2.22	0.77	1	3	-0.29	<0.001
Language and communication	2.31	0.67	1	3	2.08	0.73	1	3	-0.23	0.001
Peer engagement	2.38	0.66	1	3	1.98	0.8	1	3	-0.4	<0.001
Representation	2.31	0.57	1	3	2.01	0.69	1	3	-0.3	<0.001
	**White (all)**				**Minority ethnic group (all)**					
Assessment	1.96	0.67	1	3	1.68	0.71	1	3	-0.28	<0.001
Content	1.99	0.67	1	3	1.69	0.65	1	3	-0.3	<0.001
Culture	2.47	0.67	1	3	2.08	0.77	1	3	-0.39	<0.001
Language and communication	2.23	0.71	1	3	2.02	0.72	1	3	-0.21	<0.001
Peer engagement	2.32	0.73	1	3	1.80	0.75	1	3	-0.52	<0.001
Representation	2.34	0.63	1	3	1.78	0.61	1	3	-0.56	<0.001
	**White students**				**Minority ethnic students**					
Assessment	1.88	0.7	1	3	1.65	0.71	1	3	-0.23	0.001
Content	2.00	0.71	1	3	1.67	0.64	1	3	-0.33	<0.001
Culture	2.46	0.69	1	3	2.04	0.77	1	3	-0.42	<0.001
Language and communication	2.19	0.74	1	3	2.00	0.72	1	3	-0.19	0.006
Peer engagement	2.30	0.78	1	3	1.76	0.73	1	3	-0.54	<0.001
Representation	2.38	0.66	1	3	1.74	0.58	1	3	-0.64	<0.001
Key: * = P-values based on independent measures t-tests given the grouping of items and summation of individual item scores to create a continuous scoring scale for each domain										

## Discussion

The current study is a response to growing interest in this field. To our knowledge, it represents the first effort to explore the perceptions and experiences of stakeholders regarding decolonisation at a dental institution in the UK. Significantly, over 50% of student respondents identified themselves as from ME backgrounds. Consequently, the dataset properly represents ME students; obtaining sufficient responses from students can be challenging on some courses, especially at universities in the South West of England. Overall, average responses from ME students were lower compared to white students. These findings indicate that students from ME backgrounds may have a less optimal educational experience, with potential implications for wellbeing, attainment and subsequent employment. These results underscore the need for such audits in HE institutions to inform urgent initiatives.

There was a recognition of the need to enhance representation of all ethnicities in the curriculum by participants across the board. However, significant differences were also noted in the perceptions of staff versus students. These may be driven by several factors. First, in contrast with the student data, staff data were almost entirely made up of white respondents; only a single staff member identified themselves as from a ME background. Extrapolating the apparent trend in the student data to the staff data, it may be the predominantly white dental workforce who are not finely attuned to shortcomings in the curricula, relating to ethnicity. Additionally, the overall less favourable perceptions of students may reflect limited knowledge of positive 'hidden' practices aimed at addressing inequity. For example, in the current dental school, attainment in all assessment is closely monitored to pick up differential attainment related to demographic factors, including ethnicity. While staff are involved in these processes, students may not be aware. This raises the question whether there should be more transparency between departments and their students around practices that seek to address inequities in student experiences and outcomes. While the less favourable student results may in part reflect genuine shortcomings in the curricula, a fuller understanding of the work being undertaken by departments around these agenda may have a bearing on student perceptions. Compared to their white counterparts, ME students appear to be more sensitive to the issues related to inclusion and representation of ME groups in dental learning environments. These findings highlight that although implicit, colonial influences still persist in universities and shape the experiences and assumptions of successive generations of people from ME backgrounds. There is a need to raise awareness regarding sub-optimal experiences of ME students for a meaningful debate on ethnic disparities in dental education.

The number of institutions actively pursuing decolonisation as an institutional priority in the UK has been low in the last decade.^24^ The introduction of the Race Equality Charter by Advance HE is a positive step and apart from promoting equality, decolonisation of curricula in HE institutions is a core component of this initiative. The number of HE institutions offering staff training on decolonisation is growing following recent interventions by the Office for Students (OFS), an independent public body which was established by the Higher Education and Research Act in 2017.^7^ OFS encourages and values difference in people, thought and the provision of higher education, and predicts that a 'decolonisation agenda is on its way to becoming embedded into institutional goals'.^31^ Already, 96 universities have signed up to the Race Equality Charter by Advance HE, underscoring a growing commitment to racial equality by universities in the UK.

Indeed, findings from surveys like the current example can help raise awareness of disparities in educational experiences and outcomes relating to ethnicity and act as a catalyst for change. For example, based on the feedback by the participants in this study, our institution has identified and initiated several areas of enhancement. We now have an active equality committee in place to address issues of equality, diversity and inclusion, with representation of ME students and staff. The committee meets regularly to identify and mitigate suboptimal experiences of stakeholders. Approximately 30 students from a range of ethnic and cultural backgrounds work as equality ambassadors to champion equality across all activities at the institution. The school is now working with equality champions to organise cultural events to celebrate students and staff from diverse backgrounds, showcasing their music, dress, etc. A clear commitment to collecting data from staff and students and then sharing it in forums like these may help address the disparity in staff and students' responses to the current surveys, as discussed earlier. To demonstrate a clear commitment to equality, our university has also signed up to the Race Equality Charter of Advance HE.

Of direct relevance to the 'content' theme of the current surveys, the students and staff at our institution have created bespoke learning resources for recognition of pathological skin conditions in ME patients. These resources have received excellent feedback from students and staff across the board and are now being shared with other dental schools across the UK, demonstrating an example of good practice. The reading lists for BDS and BDTH programmes are being updated to include diverse learning resources not limited to white authors from Europe and the USA. Moreover, we are focused on developing assessment items encompassing the differential presentation of disease in ME groups, to facilitate the cultural competency of students. Prevalence of dental disease is higher while access to dental treatment and patient satisfaction rates are lower among ME populations in the UK.^20^ Enhancing the cultural competence of the future dental workforce is important to cater for the dental health needs of underserved communities in the UK and improve patient experiences. This is relevant to the 'assessment' theme from the survey. It is also hoped that these steps, related to curricular and co-curricular activities, may generate peripheral improvements to the perceptions of ME students, in terms of the themes of 'representation', 'peer engagement' and 'language and communication'. We aim to repeat the surveys in the next 12-15 months to evaluate the impact of institutional initiatives on the experiences of the stakeholders.

It is important to acknowledge limitations of the current surveys. They were devised to gain preliminary insight to staff student experiences/perspectives of decolonisation and the curriculum, in response to frontline developments (that is, a steer from the OFS to address this agenda within the institution). Further validation of the survey instrument is required with larger groups of respondents from multiple institutions. Given the data reported in this study is from a single institution, with relatively low response rates by BDS students, the findings need to be interpreted with caution due to potential effects of non-response bias and lack of generalisability. Nonetheless, to the authors' knowledge, this remains the only staff and student survey tool regarding decolonisation and the curriculum. Consequently, the current data have considerable novelty. While the current authors themselves aspire to use and refine the surveys further, other investigators are also encouraged to work with them as they see fit, be it in verbatim form, as a basis for inspiring bespoke local instruments contextualised to healthcare curricula and use in conjunction with other approaches, such as qualitative data collection.

## Conclusions

This study provides useful insights into the perceptions and experiences of students and staff regarding decolonisation of dental curriculum in two undergraduate programmes. Significant differences were noted between staff and student scores and also between white and ME students. The findings of this study underscore the need to take further steps to decolonise the dental curricula. Regular auditing by institutions appears to be an important tool for identifying factors which contribute to racial inequalities in HE. Results can then inform initiatives and practices that facilitate positive experiences for ME students and staff in their educational and work environments.

**Table:** ***Appendix 1*** Questionnaire for staff and student surveys on decolonisation of the curriculum

Theme	No.	Student wording	Staff wording
**Opening contextual questions**	I	Which department are you studying in?	Which department do you work in?
	Ii	What programme are you studying? (insert drop down of available options)	What is the main programme you support, which you will focus on when completing this audit? (insert drop down of available options)
	Iii	What year of your programme are you currently in? (insert drop down: first year/ second year/ third year/ fourth year)	
	Iv	What is your gender? (insert drop down: woman/ man/non-binary/prefer not to say)	
	v	What is your ethnic group? (insert drop down and subsequent branching question that follows exactly UK Government guidance around ethnic groups)	What is your ethnic group? (insert drop down and subsequent branching question that follows exactly UK Government guidance around ethnic groups)
**Theme 1: Representation**	1a.	Do you see yourself reflected in the examples and materials employed on the programme?	Does the programme employ examples and materials that reflect the diversity of the participants?
	1b.	If any third parties are involved in the programme (e.g. invited speakers, models, patients, employer representatives), are they drawn from diverse backgrounds?	If any third parties are involved in the programme (e.g. invited speakers, models, patients, employer representatives), are they drawn from diverse backgrounds?
	1c.	Are previous programme participants, drawn from diverse backgrounds, used to inform/ inspire you as a current student?	Are previous programme participants, drawn from diverse backgrounds, used to inform/ inspire current students?
	1d.	Are you aware of the work and accomplishments of BAME staff members in the department?	Are participants aware of the work and accomplishments of BAME staff members in the department?
	1e.	Please use the box below to add any details, which explain your responses to the questions on 'Representation'.	Please use the box below to add any details, which explain your responses to the questions on 'Representation'.
**Theme 2: Content**	2a.	Are diverse sources used in the programme (e.g. those drawn from outside Europe/ North America)?	Are diverse sources used in the programme (e.g. those drawn from outside Europe/North America)?
	2b.	Does the programme provide adequate learning opportunities for recognition of skin conditions in non-white patients In the programme, are alternative 'ways of knowing' discussed?	Does the programme provide adequate learning opportunities for recognition of skin conditions in non-white patients In the programme, are alternative 'ways of knowing' discussed?
	2d.	Is programme content made available on a flexible basis (e.g. via lecture capture)?	Is programme content made available on a flexible basis (e.g. via lecture capture)?
	2e.	Please use the box below to add any details, which explain your responses to the questions on 'Content'.	Please use the box below to add any details, which explain your responses to the questions on 'Content'.
**Theme 3:** **Peer engagement**	3a.	As part of the programme, do you get to collaborate with peers in diverse groups?	As part of the programme, do participants get to collaborate with peers in diverse groups?
	3b.	As part of the programme, are co- curricular activities relating to ethnicity and privilege promoted to you (e.g. debates, screenings, exhibitions, forums, competitions)?	As part of the programme, are co- curricular activities relating to ethnicity and privilege promoted to participants (e.g. debates, screenings, exhibitions, forums, competitions)?
	3c.	Does the programme provide you with opportunities to engage in diverse peer groups outside of scheduled classes (e.g. field trips, buddy systems, learning sets)?	Does the programme provide participants with opportunities to engage in diverse peer groups outside of scheduled classes (e.g. field trips, buddy systems, learning sets)?
	3d.	Do group discussions in the programme embrace topics related to ethnicity and privilege, even if these are uncomfortable for participants from less marginalised groups?	Do group discussions in the programme embrace topics related to ethnicity and privilege, even if these are uncomfortable for participants from less marginalised groups?
	3e.	Please use the box below to add any details, which explain your responses to the questions on 'Peer engagement'.	Please use the box below to add any details, which explain your responses to the questions on 'Peer engagement'.
**Theme 4: Assessment**	4a.	Do assessments in the programme allow you to draw in personal experiences, including those relating to ethnicity and privilege?	Do assessments in the programme allow participants to draw in personal experiences, including those relating to ethnicity and privilege?
	4b.	Do assessments in the programme feature real-world scenarios relating to ethnicity and privilege?	Do assessments in the programme feature real-world scenarios relating to ethnicity and privilege?
	4c.	In the programme, has your grasp of core content and terminology been tested using informal assessments that don't count to your final marks?	In the programme, is the participants' grasp of core content and terminology tested using informal assessments that don't count to final marks?
	4d.	Do assessments in the programme allow you to draw in latest news and current affairs relating to ethnicity and privilege?	Do assessments in the programme allow participants to draw in latest news and current affairs relating to ethnicity and privilege?
	4e.	Please use the box below to add any details, which explain your responses to the questions on 'Assessment'.	Please use the box below to add any details, which explain your responses to the questions on 'Assessment'.
**Theme 5: Language and communication**	5a.	Does the programme feature slang words, stereotypes, or language that infers the superiority of European culture and people over those from different backgrounds?	Does the programme feature slang words, stereotypes, or language that infers the superiority of European culture and people over those from different backgrounds?
	5b.	Do staff and participants on the programme master the pronunciation of all stakeholders' names?	Do staff and participants on the programme master the pronunciation of all stakeholders' names?
	5c.	Do staff and participants on the programme show micro- affirmations to individuals who share personal insights relating to ethnicity and privilege?	Do staff and participants on the programme show micro- affirmations to individuals who share personal insights relating to ethnicity and privilege?
	5d.	Are terms and acronyms from the programme summarised and shared with you in a glossary or similar format?	Are terms and acronyms from the programme summarised and shared with participants in a glossary or similar format?
	5e.	Please use the box below to add any details, which explain your responses to the questions on 'Language and communication'.	Please use the box below to add any details, which explain your responses to the questions on 'Language and communication'.
**Theme 6:** **Culture**	6a.	Do you think diversity issues are considered when learning activities on the programme are organised (e.g. when forming groups, distributing roles, managing discussions)?	Do you think diversity issues are considered when learning activities on the programme are organised (e.g. when forming groups, distributing roles, managing discussions)?
	6b.	Are BAME students on the programme made to feel different; pressurised to discuss issues of ethnicity without full consent; or made to feel that they are the only ones responsible for addressing issues relating to ethnicity and privilege?	Are BAME students on the programme made to feel different; pressurised to discuss issues of ethnicity without full consent; or made to feel that they are the only ones responsible for addressing issues relating to ethnicity and privilege?
	6c.	Have participants on the programme developed and followed rules for conduct (e.g. allowing all members to contribute; criticising ideas not individuals)?	Have participants on the programme developed and followed rules for conduct (e.g. allowing all members to contribute; criticising ideas not individuals)?
	6d.	Where challenging behaviour occurs on the programme, is it addressed head-on, and used as an opportunity to discuss issues relating to ethnicity and privilege?	Where challenging behaviour occurs on the programme, is it addressed head-on, and used as an opportunity to discuss issues relating to ethnicity and privilege?
	6e.	Please use the box below to add any details, which explain your responses to the questions on 'Culture'.	Please use the box below to add any details, which explain your responses to the questions on 'Culture'.
